# Body Oxygen Level Test (BOLT) is not associated with exercise performance in highly-trained individuals

**DOI:** 10.3389/fphys.2024.1430837

**Published:** 2024-08-28

**Authors:** Tomasz Kowalski, Kinga Rebis, Adrian Wilk, Andrzej Klusiewicz, Szczepan Wiecha, Bartłomiej Paleczny

**Affiliations:** ^1^ Department of Physiology, Institute of Sport - National Research Institute, Warsaw, Poland; ^2^ Department of Physical Education and Health in Biala Podlaska, Józef Piłsudski University of Physical Education in Warsaw, Warsaw, Poland; ^3^ Department of Physiology and Pathophysiology, Wroclaw Medical University, Wrocław, Poland

**Keywords:** breath holding, body oxygen level test, bolt, wingate, cardiopulmonary exercise test, exercise performance

## Abstract

**Introduction:**

The analysis of chemoreflex and baroreflex sensitivity may contribute to optimizing patient care and athletic performance. Breath-holding tests, such as the Body Oxygen Level Test (BOLT), have gained popularity as a feasible way to evaluate the reflex control over the cardiorespiratory system. According to its proponents, the BOLT score reflects the body’s sensitivity to carbon dioxide and homeostasis disturbances, providing feedback on exercise tolerance. However, it has not yet been scientifically validated or linked with exercise performance in highly-trained individuals. Therefore, we investigated the association of BOLT scores with the results of standard performance tests in elite athletes.

**Methods:**

A group of 49 speedskaters performed BOLT, Wingate Anaerobic Test (WAnT), and cardiopulmonary exercise test (CPET) on a cycle ergometer. Peak power, total work, and power drop were measured during WAnT. Time to exhaustion and maximum oxygen uptake were measured during CPET. Spearman’s rank correlation and multiple linear regression were performed to analyze the association of BOLT scores with parameters obtained during the tests, age, somatic indices, and training experience.

**Results:**

No significant correlations between BOLT scores and parameters obtained during WAnT and CPET were found, r(47) = −0.172–0.013, *p* = 0.248–0.984. The parameters obtained during the tests, age, somatic indices, and training experience were not significant in multiple linear regression (*p* = 0.38–0.85). The preliminary regression model showed an *R* ^2^ of 0.08 and RMSE of 9.78 sec.

**Conclusions:**

Our findings did not demonstrate a significant relationship between BOLT scores and exercise performance. Age, somatic indices, and training experience were not significant in our analysis. It is recommended to interpret BOLT concerning exercise performance in highly-trained populations with a great degree of caution.

## 1 Introduction

In recent years, there has been a significant focus on exploring the involvement of peripheral chemoreflex sensitivity (PCheS) and cardiac baroreflex sensitivity (CBaS) in various medical conditions (Kara et al., 2003; La Rovere et al., 2008) and athletic performance (Gratze et al., 2005; Gratze et al., 2008; Paleczny et al., 2021). PCheS may be defined as the ability of the body to detect and respond to changes in chemical stimuli, particularly related to oxygen and carbon dioxide (CO_2_) levels, through respiratory and cardiovascular mechanisms (Kara et al., 2003). From a clinical perspective, peripheral chemoreceptor hyperactivity is considered to be a hallmark of certain cardiovascular, respiratory, or metabolic disorders, i.e., chronic heart failure, neurogenic hypertension, obstructive sleep apnea, diabetes mellitus type II or metabolic syndrome) (Ponikowski et al., 2001; Giannoni et al., 2022). In a sports context, PCheS may also be used as a valuable tool in evaluating athletes’ performance and training needs (Ohyabu et al., 1990). CBaS refers to the ability of the baroreceptors in our body to sense changes in blood pressure and initiate appropriate cardiovascular responses to maintain homeostasis (Duffin et al., 2000). In clinical settings, CBaS analysis can provide insights into the autonomic control of blood pressure and cardiovascular function in patients with conditions such as heart failure, hypertension, and autonomic dysfunction (La Rovere et al., 2008; Parati and Ochoa, 2019). In a sports setting, CBaS analysis can be used to assess the adaptive cardiovascular responses to exercise and provide information on the athlete’s cardiovascular health, training status, and performance potential (Baumert et al., 2006; Shafiq et al., 2023). Therefore, comprehensive analysis of both chemoreflex and baroreflex sensitivity in clinical and sports settings can provide valuable information regarding respiratory and cardiovascular function and contribute to assessing, managing, and optimizing patient care and athletic performance.

The transient hypoxia tests and single-breath CO_2_ tests are widely accepted PCheS assessments. However, because of technical challenges, it is not feasible to conduct them in the field (Paleczny et al., 2021). Therefore, conducting breath-holding tests (BHT) may indicate PCheS in healthy subjects, as it presents good agreement with the aforementioned, traditionally used assessments (Trembach and Zabolotskikh, 2017). Although less associated with baroreflex assessments, BHT are reported to be helpful in BCaS prediction (Trembach and Zabolotskikh, 2018). Therefore, they represent an alternative approach that might offer insights into the state of reflex control over the cardiorespiratory system. Moreover, due to their safety and simplicity, they may be successfully used in sports settings. Multiple studies have already analyzed BHT in both clinical and applied contexts (Trembach and Zabolotskikh, 2017; Paleczny et al., 2021; Lörinczi et al., 2023).

The first research from the beginning of the 20th century regarded breath-holding and its association with altitude tolerance and predisposition to mountain sickness. Interestingly, in the 1900s large American insurance companies like New England Mutual Life or Prudential relied on BHT to evaluate health insurance risk, stimulating considerable interest in the research community and the military circles (Montoye, 1951). Consequently, breath-holding ability was used as a fitness test for Soviet parachuters. In the United States, it was investigated as an aid in escaping from damaged aircraft flying at high altitudes, as breath-holding for a long time might have helped avoid anoxia in the rarefied atmosphere. More recently, breath-holding has been investigated in respiratory disease (including COVID-19 risk stratification), distress tolerance, smoking cessation, radiation therapy or cognitive function (Hajek et al., 1987; Kellett and Mullan, 2002; Zavoreo et al., 2010; Sütterlin et al., 2013; Boda-Heggemann et al., 2016; Messineo et al., 2021). However, research in high-performance sports remains scarce.

The Body Oxygen Level Test (BOLT) is one of the most recognized BHT. According to its proponents, the BOLT score reflects the body’s sensitivity to CO_2_ and homeostasis disturbances, providing feedback regarding functional breathing and exercise tolerance (McKeown, 2021). It was popularized by Patrick McKeown and “The Oxygen Advantage^®^,” which is a breathwork method aiming to optimize health, mental clarity, and performance (McKeown, 2015). Despite limited scientific rationale, “The Oxygen Advantage^®^” is frequently mentioned in thematic literature, personal training websites, YouTube videos, podcasts, and remains a fundamental part of breathwork workshops all over the world. As of July 2024, Google Search finds around 205,000,000 websites associated with the “Body Oxygen Level Test.”

However, BOLT has not yet been scientifically validated in sports settings. Therefore, we investigated the association of BOLT scores with well-established performance tests, age, somatic indices, and training experience in well-trained individuals. The study focuses on exercise tolerance in severe and extreme intensity domains, as they provoke significant homeostatic perturbations (Korzeniewski and Rossiter, 2022).

## 2 Methods and materials

The study has been carried out according to the guidelines for observational studies presented by the EQUATOR Network in the Strengthening the Reporting of Observational Studies in Epidemiology (STROBE) Statement (von elm et al., 2007). All the study procedures have been performed in accordance with the Declaration of Helsinki. The study protocol was reviewed and approved by the Institute of Sport - National Research Institute Ethics Committee (approval no KEBN-23-91-AW). The participants were informed about the applied procedures in oral and written forms. The written consent was obtained from all the study participants. The participants were free to withdraw from the study at any time without further consequences. The study has been registered at ClinicalTrials.gov under NCT06236490.

### 2.1 Participants

**FIGURE 1:**
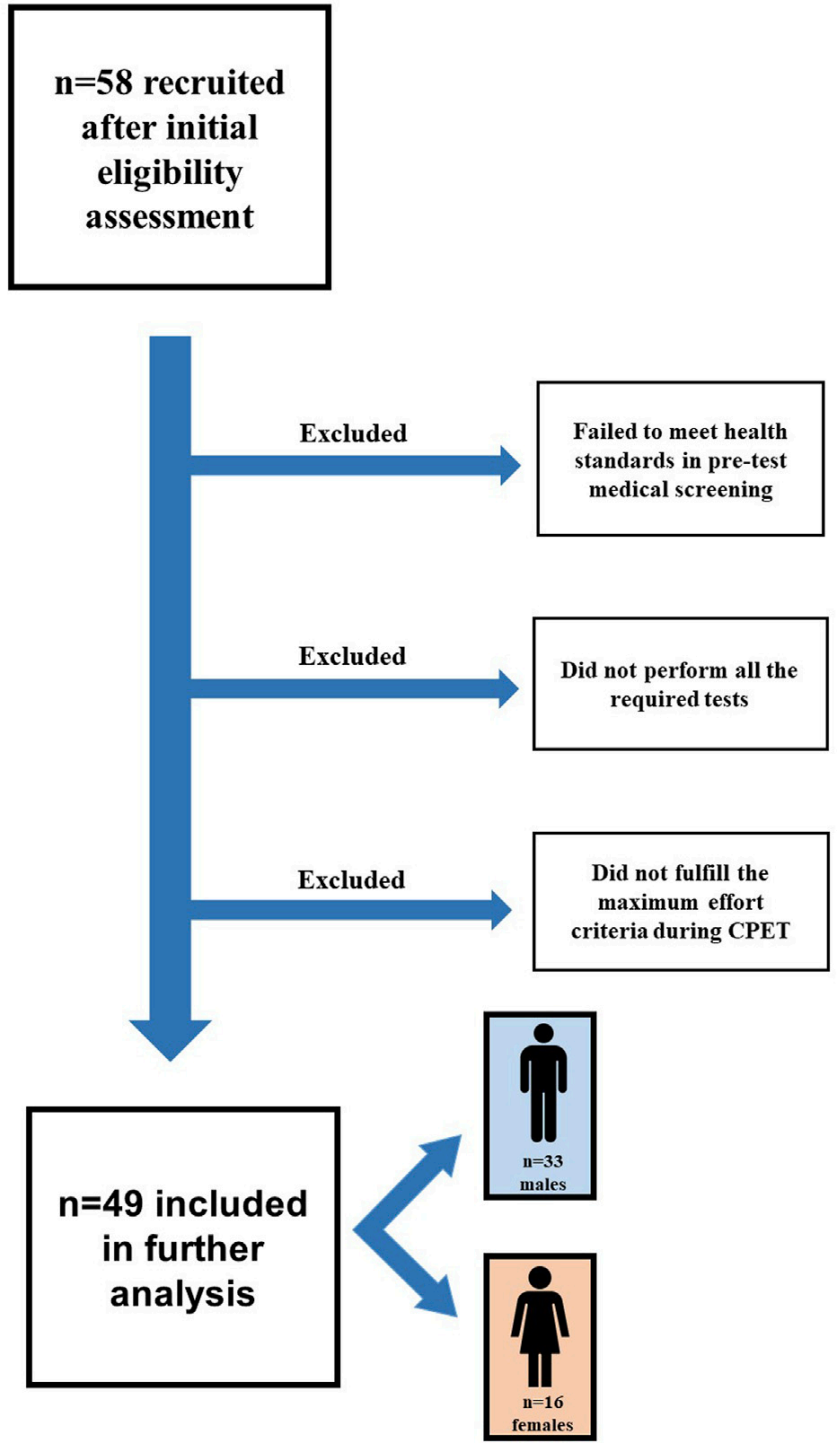
The flow diagram for the detailed recruitment process. CPET - cardiopulmonary exercise testing.

The investigated group consisted of 49 well-trained speedskaters (n = 16 females, n = 33 males) recruited with convenience sampling between June and September 2023. All the study participants represented either elite or development national teams from three different countries. The participants competed in either short-track or long-track sprint and medium-distance events. Inclusion criteria were as follows: a valid medical certificate for competitive speedskating, a minimum of 4 years of competitive training, and no prior exposure to respiratory or respiratory muscle training. Exclusion criteria were as follows: any chronic or acute medical condition within the last 4 weeks, recent stay at an altitude over 1,500 m above sea level within the last 4 weeks, and the use of any ongoing medications, smoking, any ongoing allergy. The required sample size was calculated with G* Power (version 3.1.9.2; Germany), with effect size ƒ^2^ = 0.50, level of significance set at α = 0.05, power (1 − β) = 0.80, and number of predictors = 9 (Linear multiple regression: Fixed model, *R* ^2^ deviation from zero). According to the calculations, the required total sample size was 41 participants. In our study, 58 subjects were recruited to account for possible dropouts. Finally, 49 participants completed all the required procedures and were included in further analysis. The flow diagram for the detailed recruitment process is presented in Figure 1. The body height was measured with the free-standing digital stadiometer seca 274 (seca GmbH and Co. KG., Hamburg, Germany). The body composition was assessed before breakfast with the bioelectrical impedance analysis system Tanita BC-420MA (Tanita Corporation, Tokyo, Japan). The participants’ characteristics are presented in Table 1.

**TABLE 1:** The participants’ characteristics.

Variable/Group	Females (n = 16)	Males (n = 33)
Age (years)	19.5 ± 4.1	19.1 ± 2.3
Body mass (kg)	61.0 ± 6.0	71.7 ± 8.7
Body height (cm)	169.4 ± 6.4	178.4 ± 6.4
Body fat (%)	17.5 ± 4.0	8.5 ± 3.0
VO_2_max (mL·kg^−1^ min^−1^)	49.5 ± 4.8	59.5 ± 5.1
Training experience (years)	8.8 ± 4.9	8.6 ± 4.1

Values are mean ± standard deviation. Abbreviations: VO_2_max, maximum oxygen uptake.

### 2.2 Testing procedure

**FIGURE 2:**
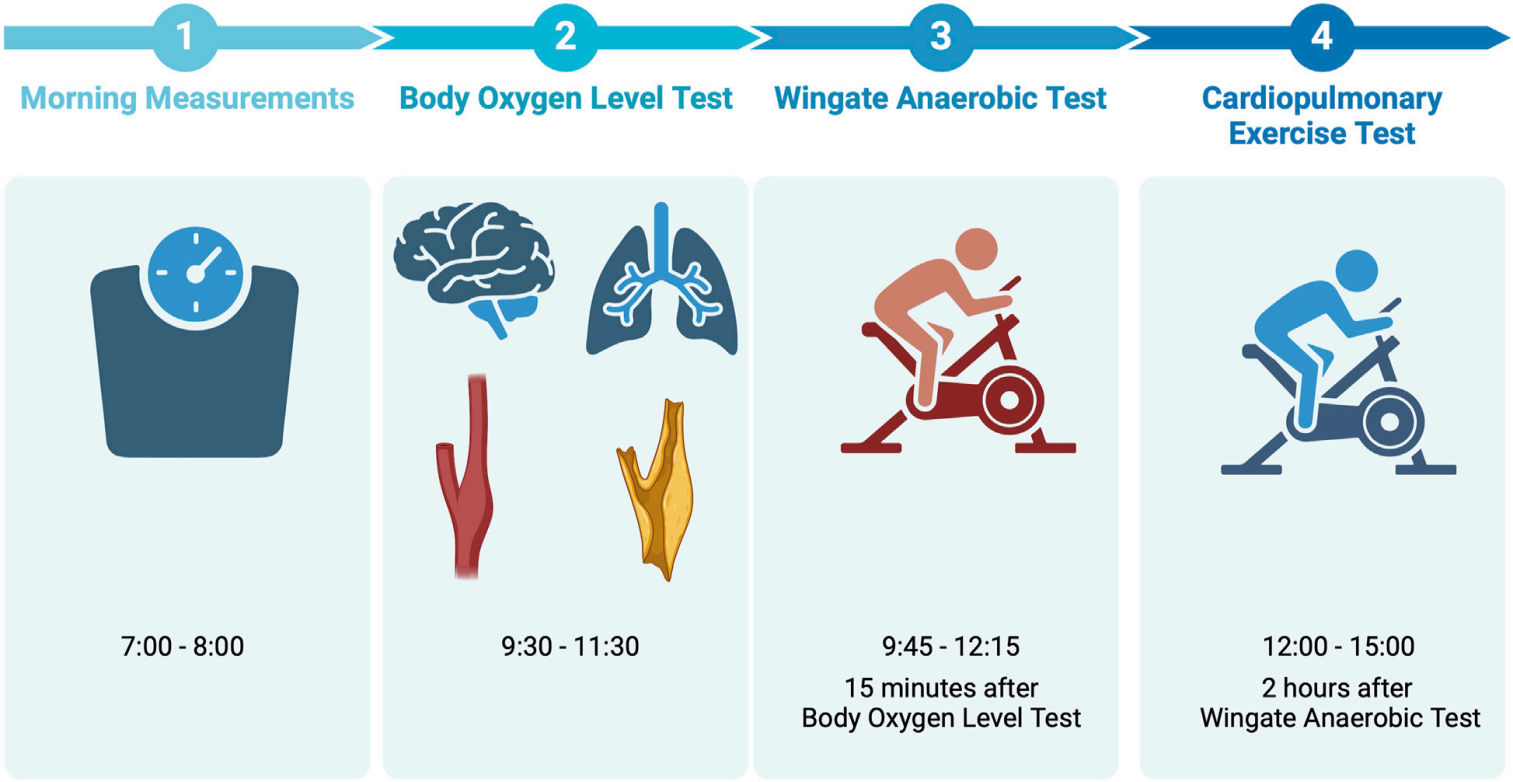
The timeline of the testing day.

All the testing procedures were conducted on the same day at the Institute of Sport - National Research Institute (Warsaw, Poland) or its temporary field station (Gdansk, Poland) between June and September 2023. All the study participants were familiar with the performance testing procedures, as they have performed both the Wingate Anaerobic Test (WAnT) and the cardiopulmonary exercise test (CPET) multiple times before, in the same settings and with the same protocols. The participants were advised to avoid strenuous physical activity for 2 days before the testing. BOLT took place between 9:30 and 11:30, WAnT took place between 9:45 and 12:15, CPET took place between 12:00 and 15:00. The break between BOLT and WAnT took 15 min, the break between WAnT and CPET took 2 h. The research team performing WAnT and CPET was blinded to the participants’ BOLT scores. The timeline of the testing day is presented in Figure 2.

**FIGURE 3:**
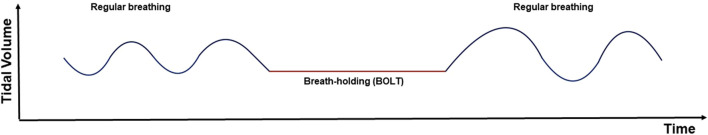
The visual presentation of breathing pattern around the Body Oxygen Level Test (BOLT).

BOLT was performed according to “The Oxygen Advantage^®^” guidelines (McKeown, 2015), which allow for the assessment reliability (Lörinczi et al., 2023). The study participants sat for 15 min before measuring the BOLT score. During this time they were carefully instructed on how to perform the measurement. It was underlined that BOLT is a subjective and submaximal assessment. The participants were informed that we do not measure how long one can maximally hold their breath until the breaking point, but the time it takes for the body to react to lack of air and feel the first desire to breathe. After a few normal breaths through the nose, after the exhalation, the participants held their breath until they felt the first definite desire to breathe or felt the first involuntary contractions of the respiratory muscles. The first was assessed by the participants, the latter was also visually assessed by the researchers. The nose clip was applied to prevent any gas exchange. The researchers measured the time with a Finis 3x300M stopwatch (Finis USA, Livermore, CA, United States). Time in seconds, to one decimal place, was noted as a BOLT score. The visualization of BOLT is presented in Figure 3.

WAnT was conducted with the Monark 874E Cycle Ergometer (Monark Exercise AB, Sweden). After a standardized warm-up, the participants underwent a 30-second WAnT with resistance set at 7.5% of the individual body mass. They were instructed to attain the highest peak power output as quickly as possible and maintain the highest power output throughout the entire duration of the test. The participants received motivating and enthusiastic verbal support. The following indices were measured and included in the further analysis: peak power (PP), total work (TW), and power drop (DP). All the indices were calculated with the dedicated software (MCE 6.0, JBA Z. Staniak, Poland) linked to the cycle ergometer.

CPET was performed with the Cortex Metamax B3 (Cortex Biophysik GmbH, Leipzig, Germany), breath-by-breath method, and the Cyclus II Ergometer (RBM, Leipzig, Germany). The testing equipment has been calibrated according to the manufacturer’s instructions. Participants underwent an incremental ramp test, commencing at 55–70 W and incrementally increasing the load by 0.17–0.28 W·sec^−1^, individually adjusted for body mass and fitness status. The participants were instructed to continue the effort until total exhaustion. To be included in further analysis, the participants had to fulfill at least five out of the six maximum effort criteria (Wiecha et al., 2023) as follows: 1) respiratory exchange ratio ≥1.10, 2) present oxygen uptake plateau (growth <100 mL·min^–1^ in oxygen uptake despite an increase in workload), 3) respiratory frequency ≥ 45 breaths·min^–1^, 4) declared subjective exertion intensity during CPET ≥18 according to the Borg Scale (Borg, 1998), 5) blood lactate concentration ≥8 mmol·L^–1^, 6) peak heart rate ≥15 beats·min^–1^ below maximal heart rate predicted according to Lach et al. (2021). The following indices were measured with 15-second averages and included in further analysis: maximum oxygen uptake (VO_2_max) in mL·min⁻^1^·kg⁻^1^, and time from achieving VO_2_max to the cessation of the test in seconds.

### 2.3 Statistical analysis

The Shapiro-Wilk test and visual analysis of plot figures were applied to assess the normality of data distribution. Spearman’s rank correlation was performed to assess the strength and direction of the relationship between BOLT scores and the results of performance tests. Multiple linear regression was employed to analyze the association of BOLT score with parameters obtained during the tests, age, somatic indices, and training experience. A traditional level of significance (*p* < 0.05) was used. The Durbin-Watson test was used to detect the presence of autocorrelation between the residuals from the regression analysis. The statistical analyses were performed with the JASP Team statistical package (JASP, Amsterdam, Netherlands, Version 0.17.2). The correlation visualization was prepared with GraphPad Prism (GraphPad Software, San Diego, CA, United States, version 10.1.2).

## 3 Results

**FIGURE 4:**
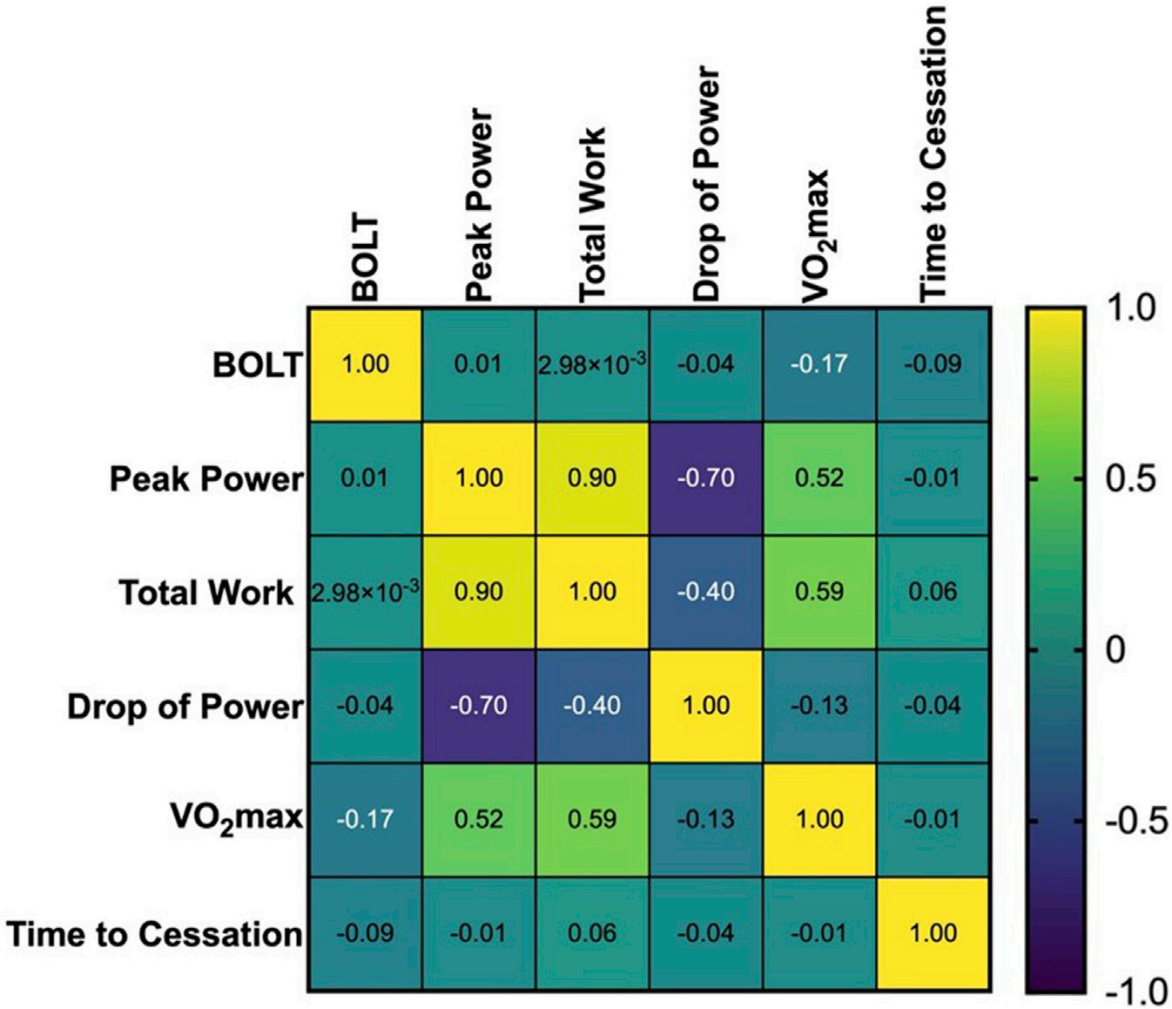
The correlation between BOLT scores and parameters obtained during WAnT and CPET.

No significant correlations between BOLT scores and parameters obtained during WAnT and CPET were found, r(47) = −0.172 to 0.013, *p* = 0.248 to 0.984. The correlation matrix is presented in Figure 4.

After the correlation analysis, TW was excluded from the follow-up regression test, as a strong correlation between PP and TW was found. The included data met the assumption of independent errors (Durbin-Watson value = 1.924). The parameters obtained during the tests, age, somatic indices, and training experience were not significant in multiple linear regression. The top-performing preliminary regression model (see Table 2) showed an *R* ^2^ of only 0.08 [F(8,41) = 0.429, *p* = 0.896] and RMSE of 9.78 sec.

**TABLE 2:** Results for the top-performing preliminary regression model.

	Coefficient	Standard error	t-value	*p*-value
Age (years)	−0.717	0.983	−0.729	0.470
Body mass (kg)	−0.306	0.355	−0.863	0.394
Body height (cm)	0.360	0.429	0.839	0.407
Peak power (W/kg)	1.225	2.553	0.480	0.634
Power drop (W/kg/s)	11.929	60.555	0.197	0.845
VO_2_max (mL·kg⁻^1^ min⁻^1^)	−0.237	0.270	−0.881	0.384
Time to cessation (seconds)	−0.007	0.027	−0.262	0.795
Training experience (years)	0.169	0.658	0.257	0.799

Abbreviations: VO_2_max, maximum oxygen uptake.

## 4 Discussion

Although the debate regarding the usefulness of BHT has been going on for over 50 years, this might be the first study carried out in an elite cohort of highly-trained individuals. Our findings do not confirm a significant relationship between BOLT scores and exercise performance in such a population. Moreover, age, somatic indices, and training experience were not significantly associated with BOLT scores in our analysis. The results of our study suggest that BOLT scores should not be interpreted concerning exercise performance in elite athletes. The source of the lack of relationship between BOLT and investigated variables remains to be established. Speculatively, it may be associated with BOLT’s limitations, narrow variability of PCheS and CBaS in elite athletes, both, or other factors.

The utility of the BHT in patients or in distinguishing the sick from the healthy was proven in multiple studies (Viecili et al., 2012; Nannini et al., 2007; Hedhli et al., 2021). The studies regarding patients investigated the association of PCheS and CBaS with exercise tolerance, presenting mixed results (Schultz and Sun, 2000; Barnai et al., 2005; Phillips et al., 2019). Noteworthy, the athletic population exhibits different physiological characteristics compared to patients or the typical healthy population (Petek et al., 2022; Kasiak et al., 2024). However, the authors have not found congenial research in the population of elite athletes. The presented results are coherent with research evaluating breath-holding training and its influence on athletic performance. A systematic review and meta-analysis presented by de Asis-Fernandez (2022) found no influence of breath-holding training on VO_2_max (de Asís-Fernández et al., 2022). Multiple studies in healthy and athletic populations report a lack of significant effects of apnea training on exercise performance, whilst improving breath-holding capacity (Lemaître et al., 2009; Son et al., 2020; Bouten et al., 2022). These findings suggest no link between breath-holding capacity and exercise performance, as changes in the first do not influence the latter.

A recent study from Paleczny et al. (2021) linked BHT results with faster marathon performance and higher VO_2_max in healthy males aged above 50 years old. Both indices of individual performance employed in the study, marathon time and VO_2_max as assessed with the treadmill CPET, were correlated to the BHT result. Athletes exhibiting high exercise capability (as evidenced by shorter marathon completion time, and higher VO_2_max) were characterized by a longer duration of voluntary apnea measured to the onset of reflex contractions of the diaphragm. Noteworthy, similar correlations with individual performance indices were found for another well-established measure of PChS, namely the hypoxic ventilatory response. Factors underlying such discrepancy between the current study and the report by Paleczny et al. remain unknown but may include patients’ characteristics (>35 years age gap between the populations studied, and different gender distribution), and methodological differences regarding the endpoint of BHT apnea (i.e., contractions of the diaphragm/respiratory muscles as assess with palpation in the study by Paleczny et al. or visually in the current study).

Lörinczi et al. (2023) underlined the necessity of developing higher resistance to excessive CO_2_ in athletes, as naturally linked to the PCheS and CBaS. They recalled that athletes with lower CO_2_ tolerance tend to shift quickly to mouth breathing and dysfunctional breathing patterns when exercise intensity increases. Indeed, BHT have been found useful in diagnosing dysfunctional breathing (Kiesel et al., 2017), which is prevalent in well-trained endurance athletes (Sikora et al., 2024). The association of CO_2_ tolerance with mouth breathing may be found in the literature (Gilbert et al., 2014; McKeown, 2021), although the mechanistic evidence behind this connection is limited. Nevertheless, mouth breathing and hyperventilation are linked to faster muscle fatigue, increased water loss, and higher asthma or exercise-induced bronchoconstriction risk (Gilbert, 1999; Svensson et al., 2006; Boulet and O’Byrne, 2015). These are important factors when managing the training process, in both performance and athlete’s health contexts. However, this indirect influence of breathing patterns was not significant enough to affect the results of WAnT and CPET in our study.

Numerous techniques and commercial products related to respiratory health are now promoted with claims of therapeutic or performance-enhancing benefits. However, many of such claims lack the scientific evidence that substantiates them, creating a significant research gap and a need for rigorous research (Illidi et al., 2023). In the context of our study, the association of BOLT scores with exercise performance requires further investigation in different populations and using different testing modalities. Additionally, monitoring breathing characteristics during exercise tests and its association with BHT might provide useful insight. Furthermore, the link between BOLT scores and actual physiological occurrences associated with baroreceptor and chemoreceptor function remains to be established. Moreover, BOLT itself requires developing an appropriate methodology to ensure the validity of the assessment. The metrological properties such as reproducibility and robustness should be taken into consideration.

The study is not free from limitations. We investigated a homogeneous group of well-trained speedskaters. Due to the unique group characteristics, the reported findings may not be applicable to different populations. Moreover, the lack of participants’ familiarity with BOLT may be considered the limitation of the study, as the repeatability of measurement and self-reporting on diaphragm contractions are not thoroughly investigated. Furthermore, we did not track the menstrual cycle phase or severity of premenstrual syndrome in female athletes. The menstrual cycle phase might influence CO_2_ sensitivity and maximum breath-holding time (Dutton et al., 1989; Cherouveim et al., 2020). Severe forms of premenstrual syndrome are associated with CO_2_ hypersensitivity (Nillni et al., 2017). Therefore, both might influence BOLT results.

## 5 Conclusion

It is recommended to interpret BOLT concerning exercise performance in well-trained individuals with a great degree of caution. Whether the lack of relationship between BOLT and exercise performance is associated with the test’s limitations, narrow variability of PCheS and CBaS in elite athletes, both, or other factors, remains to be established.

## Data Availability

The raw data supporting the conclusions of this article will be made available by the authors, without undue reservation.

## References

[B1] Barnai M. Laki I. Gyurkovits K. Angyan L. Horvath G. (2005). Relationship between breath-hold time and physical performance in patients with cystic fibrosis. Eur. J. Appl. Physiol. 95, 172–178. 10.1007/s00421-005-1350-3 16007450

[B2] Baumert M. Brechtel L. Lock J. Hermsdorf M. Wolff R. Baier V. (2006). Heart rate variability, blood pressure variability, and baroreflex sensitivity in overtrained athletes. Clin. J. Sport Med. 16, 412–417. 10.1097/01.jsm.0000244610.34594.07 17016118

[B3] Boda-Heggemann J. Knopf A.-C. Simeonova-Chergou A. Wertz H. Stieler F. Jahnke A. (2016). Deep inspiration breath hold-based radiation therapy: a clinical review. Int. J. Radiat. Oncol. Biol. Phys. 94, 478–492. 10.1016/j.ijrobp.2015.11.049 26867877

[B4] Borg G. (1998). Borg’s perceived exertion and pain scales. Human Kinetics. Available at: https://psycnet.apa.org/fulltext/1998-07179-000.pdf. 104

[B5] Boulet L.-P. O’Byrne P. M. (2015). Asthma and exercise-induced bronchoconstriction in athletes. N. Engl. J. Med. 372, 641–648. 10.1056/NEJMra1407552 25671256

[B6] Bouten J. Debusschere J. Lootens L. Declercq L. Van Eenoo P. Boone J. (2022). Six weeks of static apnea training does not affect Hbmass and exercise performance. J. Appl. Physiol. 132, 673–681. 10.1152/japplphysiol.00770.2021 35050796

[B7] Cherouveim E. D. Botonis P. G. Tsakiris T. Koskolou M. D. Geladas N. D. (2020). The effect of menstrual cycle on maximal breath-hold time. Respir. Physiol. Neurobiol. 274, 103381. 10.1016/j.resp.2020.103381 31923591

[B8] de Asís-Fernández F. Sereno D. Turner A. P. González-Mohíno F. González-Ravé J. M. (2022). Effects of apnoea training on aerobic and anaerobic performance: a systematic review and meta-analysis. Front. Physiol. 13, 964144. 10.3389/fphys.2022.964144 36237527 PMC9551563

[B9] Duffin J. Mohan R. M. Vasiliou P. Stephenson R. Mahamed S. (2000). A model of the chemoreflex control of breathing in humans: model parameters measurement. Respir. Physiol. 120, 13–26. 10.1016/s0034-5687(00)00095-5 10786641

[B10] Dutton K. Blanksby B. A. Morton A. R. (1989). CO_2_ sensitivity changes during the menstrual cycle. J. Appl. Physiol. 67, 517–522. 10.1152/jappl.1989.67.2.517 2507498

[B11] Giannoni A. Gentile F. Buoncristiani F. Borrelli C. Sciarrone P. Spiesshoefer J. (2022). Chemoreflex and baroreflex sensitivity hold a strong prognostic value in chronic heart failure. JACC Heart Fail 10, 662–676. 10.1016/j.jchf.2022.02.006 36049816

[B12] Gilbert C. (1999). Hyperventilation and the body. Accid. Emerg. Nurs. 7, 130–140. 10.1016/s0965-2302(99)80072-1 10693382

[B13] Gilbert C. Chaitow L. Bradley D. (2014). Recognizing and treating breathing disorders: a multidisciplinary approach. Elsevier Health Sciences. Available at: https://play.google.com/store/books/details?id=GYf6AQAAQBAJ.

[B14] Gratze G. Mayer H. Luft F. C. Skrabal F. (2008). Determinants of fast marathon performance: low basal sympathetic drive, enhanced postcompetition vasodilatation and preserved cardiac performance after competition. Br. J. Sports Med. 42, 882–888. 10.1136/bjsm.2007.044271 18203868

[B15] Gratze G. Rudnicki R. Urban W. Mayer H. Schlögl A. Skrabal F. (2005). Hemodynamic and autonomic changes induced by Ironman: prediction of competition time by blood pressure variability. J. Appl. Physiol. 99, 1728–1735. 10.1152/japplphysiol.00487.2005 16002770

[B16] Hajek P. Belcher M. Stapleton J. (1987). Breath-holding endurance as a predictor of success in smoking cessation. Addict. Behav. 12, 285–288. 10.1016/0306-4603(87)90041-4 3661283

[B17] Hedhli A. Slim A. Ouahchi Y. Mjid M. Koumenji J. Cheikh Rouhou S. (2021). Maximal voluntary breath-holding tele-inspiratory test in patients with chronic obstructive pulmonary disease. Am. J. Mens. Health 15, 15579883211015857. 10.1177/15579883211015857 33993797 PMC8127757

[B18] Illidi C. R. Romer L. M. Johnson M. A. Williams N. C. Rossiter H. B. Casaburi R. (2023). Distinguishing science from pseudoscience in commercial respiratory interventions: an evidence-based guide for health and exercise professionals. Eur. J. Appl. Physiol. 123, 1599–1625. 10.1007/s00421-023-05166-8 36917254 PMC10013266

[B19] Kara T. Narkiewicz K. Somers V. K. (2003). Chemoreflexes--physiology and clinical implications. Acta Physiol. Scand. 177, 377–384. 10.1046/j.1365-201X.2003.01083.x 12609009

[B20] Kasiak P. Kowalski T. Rębiś K. Klusiewicz A. Ładyga M. Sadowska D. (2024). Is the ventilatory efficiency in endurance athletes different? findings from the NOODLE study. J. Clin. Med. Res. 13, 490. 10.3390/jcm13020490 PMC10816682 38256624

[B21] Kellett C. Mullan J. (2002). Breathing control techniques in the management of asthma. Physiotherapy 88, 751–758. 10.1016/S0031-9406(05)60719-5

[B22] Kiesel K. Rhodes T. Mueller J. Waninger A. Butler R. (2017). Development of a screening protocol to identify individuals with dysfunctional breathing. Int. J. Sports Phys. Ther. 12, 774–786. Available at: https://www.ncbi.nlm.nih.gov/pubmed/29181255. 29181255 PMC5685417

[B23] Korzeniewski B. Rossiter H. B. (2022). Skeletal muscle biochemical origin of exercise intensity domains and their relation to whole-body V̇O_2_ kinetics. Biosci. Rep. 42. 10.1042/BSR20220798 PMC9366749 35880531

[B24] Lach J. Wiecha S. Śliż D. Price S. Zaborski M. Cieśliński I. (2021). HR max prediction based on age, body composition, fitness level, testing modality and sex in physically active population. Front. Physiol. 12, 695950. 10.3389/fphys.2021.695950 34393819 PMC8362801

[B25] La Rovere M. T. Pinna G. D. Raczak G. (2008). Baroreflex sensitivity: measurement and clinical implications. Ann. Noninvasive Electrocardiol. 13, 191–207. 10.1111/j.1542-474X.2008.00219.x 18426445 PMC6931942

[B26] Lemaître F. Seifert L. Polin D. Juge J. Tourny-Chollet C. Chollet D. (2009). Apnea training effects on swimming coordination. J. Strength Cond. Res. 23, 1909–1914. 10.1519/JSC.0b013e3181b073a8 19675466

[B27] Lörinczi F. Lörincziová D. Vanderka M. (2023). Reliability of breath-holding tests with potential for use in sports practice. JoKeS 33, 27–34. 10.5604/01.3001.0053.9000

[B28] McKeown P. (2015). The Oxygen advantage: the simple, scientifically proven breathing technique that will revolutionise your health and fitness. Hachette UK. Available at: https://play.google.com/store/books/details?id=7FPXBAAAQBAJ.

[B29] McKeown P. (2021) The breathing cure: exercises to develop new breathing habits for a healthier, happier and longer life. OxyAt Books.

[B30] Messineo L. Perger E. Corda L. Joosten S. A. Fanfulla F. Pedroni L. (2021). Breath-holding as a novel approach to risk stratification in COVID-19. Crit. Care 25, 208. 10.1186/s13054-021-03630-5 34127052 PMC8200551

[B31] Montoye H. J. (1951). Breath-holding as a measure of physical fitness. Res. Q. Am. Assoc. Health Phys. Educ. Recreat. 22, 356–376. 10.1080/10671188.1951.10621326

[B32] Nannini L. J. Zaietta G. A. Guerrera A. J. Varela J. A. Fernández O. M. Flores D. M. (2007). Breath-holding test in subjects with near-fatal asthma. A new index for dyspnea perception. Respir. Med. 101, 246–253. 10.1016/j.rmed.2006.05.013 16824744

[B33] Nillni Y. I. Pineles S. L. Rohan K. J. Zvolensky M. J. Rasmusson A. M. (2017). The influence of the menstrual cycle on reactivity to a CO_2_ challenge among women with and without premenstrual symptoms. Cogn. Behav. Ther. 46, 239–249. 10.1080/16506073.2016.1236286 27687294 PMC6598439

[B34] Ohyabu Y. Usami A. Ohyabu I. Ishida Y. Miyagawa C. Arai T. (1990). Ventilatory and heart rate chemosensitivity in track-and-field athletes. Eur. J. Appl. Physiol. Occup. Physiol. 59, 460–464. 10.1007/BF02388629 2303052

[B35] Paleczny B. Seredyński R. Wyciszkiewicz M. Nowicka-Czudak A. Łopusiewicz W. Adamiec D. (2021). Low ventilatory responsiveness to transient hypoxia or breath-holding predicts fast marathon performance in healthy middle-aged and older men. Sci. Rep. 11, 10255. 10.1038/s41598-021-89766-4 33986451 PMC8119959

[B36] Parati G. Ochoa J. E. (2019). Prognostic value of baroreflex sensitivity in heart failure. A 2018 reappraisal. Eur. J. Heart Fail 21, 59–62. 10.1002/ejhf.1334 30468274

[B37] Petek B. J. Tso J. V. Churchill T. W. Guseh J. S. Loomer G. DiCarli M. (2022). Normative cardiopulmonary exercise data for endurance athletes: the cardiopulmonary health and endurance exercise registry (CHEER). Eur. J. Prev. Cardiol. 29, 536–544. 10.1093/eurjpc/zwab150 34487164

[B38] Phillips D. B. Collins S. É. Bryan T. L. Wong E. Y. L. McMurtry M. S. Bhutani M. (2019). The effect of carotid chemoreceptor inhibition on exercise tolerance in chronic obstructive pulmonary disease: a randomized-controlled crossover trial. Respir. Med. 160, 105815. 10.1016/j.rmed.2019.105815 31739245

[B39] Ponikowski P. Chua T. P. Anker S. D. Francis D. P. Doehner W. Banasiak W. (2001). Peripheral chemoreceptor hypersensitivity: an ominous sign in patients with chronic heart failure. Circulation 104, 544–549. 10.1161/hc3101.093699 11479251

[B40] Schultz H. D. Sun S. Y. (2000). Chemoreflex function in heart failure. Heart fail. Rev. 5, 45–56. 10.1023/A:1009846123893 16228915

[B41] Shafiq M. A. Ellingson C. A. Krätzig G. P. Dorsch K. D. Neary J. P. Singh J. (2023). Differences in heart rate variability and baroreflex sensitivity between male and female athletes. J. Clin. Med. Res. 12, 3916. 10.3390/jcm12123916 PMC10299049 37373610

[B42] Sikora M. Mikołajczyk R. Łakomy O. Karpiński J. Żebrowska A. Kostorz-Nosal S. (2024). Influence of the breathing pattern on the pulmonary function of endurance-trained athletes. Sci. Rep. 14, 1113. 10.1038/s41598-024-51758-5 38212427 PMC10784475

[B43] Son H. Jeon Y. Kim H. Son H. Jeon Y. Kim H. (2020). Effects of static apnea training on pulmonary function, blood lactate response and exercise performance of elite swimmers. Exerc Sci. 29, 272–280. 10.15857/ksep.2020.29.3.272

[B44] Sütterlin S. Schroijen M. Constantinou E. Smets E. Van den Bergh O. Van Diest I. (2013). Breath holding duration as a measure of distress tolerance: examining its relation to measures of executive control. Front. Psychol. 4, 483. 10.3389/fpsyg.2013.00483 23908639 PMC3725515

[B45] Svensson S. Olin A. C. Hellgren J. (2006). Increased net water loss by oral compared to nasal expiration in healthy subjects. Rhinology 44, 74–77. Available at: https://www.ncbi.nlm.nih.gov/pubmed/16550955. 16550955

[B46] Trembach N. Zabolotskikh I. (2017). Breath-holding test in evaluation of peripheral chemoreflex sensitivity in healthy subjects. Respir. Physiol. Neurobiol. 235, 79–82. 10.1016/j.resp.2016.10.005 27756650

[B47] Trembach N. Zabolotskikh I. (2018). Arterial baroreflex sensitivity: relationship with peripheral chemoreflex in patients with chronic heart failure. Artery Res. 24, 9–15. 10.1016/j.artres.2018.10.002

[B48] Viecili R. B. Silva D. R. Sanches P. R. S. Müller A. F. Silva D. P. (2012). Real-time measurement of maximal voluntary breath-holding time in patients with obstructive ventilatory defects and normal controls. J. Pulmon. Resp. Med. 2(5), 1–3. 10.4172/2161-105X.1000127

[B49] von Elm E. Altman D. G. Egger M. Pocock S. J. Gøtzsche P. C. Vandenbroucke J. P. (2007). The Strengthening the Reporting of Observational Studies in Epidemiology (STROBE) statement: guidelines for reporting observational studies. Ann. Intern. Med. 147, 573–577. 10.7326/0003-4819-147-8-200710160-00010 17938396

[B50] Wiecha S. Kasiak P. S. Szwed P. Kowalski T. Cieśliński I. Postuła M. (2023). VO2max prediction based on submaximal cardiorespiratory relationships and body composition in male runners and cyclists: a population study. Elife 12, e86291. 10.7554/eLife.86291 37162318 PMC10198721

[B51] Zavoreo I. Kes V. B. Morović S. Serić V. Demarin V. (2010). Breath holding index in detection of early cognitive decline. J. Neurol. Sci. 299, 116–119. 10.1016/j.jns.2010.08.062 20884013

